# Characterization of Bioinks Prepared via Gelifying Extracellular Matrix from Decellularized Porcine Myocardia

**DOI:** 10.3390/gels9090745

**Published:** 2023-09-13

**Authors:** Héctor Sanz-Fraile, Carolina Herranz-Diez, Anna Ulldemolins, Bryan Falcones, Isaac Almendros, Núria Gavara, Raimon Sunyer, Ramon Farré, Jorge Otero

**Affiliations:** 1 Unitat de Biofísica i Bioenginyeria, Facultat de Medicina i Ciències de la Salut, Universitat de Barcelona, 08036 Barcelona, Spain; hector.sanz.fraile@hotmail.com (H.S.-F.); carolinaherranz@ub.edu (C.H.-D.); anna.ulldemolins@ub.edu (A.U.); bfalco86@gmail.com (B.F.); ialmendros@ub.edu (I.A.); ngavara@ub.edu (N.G.); rsunyer@ub.edu (R.S.); rfarre@ub.edu (R.F.); 2 CIBER de Enfermedades Respiratorias, 28029 Madrid, Spain; 3 Institut d’Investigacions Biomèdiques August Pi i Sunyer, 08036 Barcelona, Spain; 4 The Institute for Bioengineering of Catalonia (IBEC), 08028 Barcelona, Spain; 5 The Barcelona Institute of Science and Technology (BIST), 08036 Barcelona, Spain; 6 Institute of Nanoscience and Nanotechnology (IN2UB), Universitat de Barcelona, 08028 Barcelona, Spain; 7 CIBER de Bioingeniería, Biomateriales y Nanomedicina, 28029 Madrid, Spain

**Keywords:** 3D bioprinting, hydrogels, extracellular matrix, decellularized cardiac tissue, biomaterials, mesenchymal stromal cells

## Abstract

Since the emergence of 3D bioprinting technology, both synthetic and natural materials have been used to develop bioinks for producing cell-laden cardiac grafts. To this end, extracellular-matrix (ECM)-derived hydrogels can be used to develop scaffolds that closely mimic the complex 3D environments for cell culture. This study presents a novel cardiac bioink based on hydrogels exclusively derived from decellularized porcine myocardium loaded with human-bone-marrow-derived mesenchymal stromal cells. Hence, the hydrogel can be used to develop cell-laden cardiac patches without the need to add other biomaterials or use additional crosslinkers. The scaffold ultrastructure and mechanical properties of the bioink were characterized to optimize its production, specifically focusing on the matrix enzymatic digestion time. The cells were cultured in 3D within the developed hydrogels to assess their response. The results indicate that the hydrogels fostered inter-cell and cell-matrix crosstalk after 1 week of culture. In conclusion, the bioink developed and presented in this study holds great potential for developing cell-laden customized patches for cardiac repair.

## 1. Introduction

There is a significant lack of organs for transplantation and replacements for implants and this problem will increase with the population aging, particularly in developed countries. Tissue engineering and regenerative medicine have emerged as potential solutions to overcome the progressive reduction of viable organ donors [1]. In the specific case of the heart, engineering implantable hearts built in the laboratory is still far away [2]. Hence, novel constructs based on decellularized tissue [3] or electroconductive scaffolds [4] have been recently developed to produce re-cellularized grafts (in the form of cardiac patches) to ameliorate the function of diseased hearts [5]. Several synthetic materials have been used to develop these scaffolds, but they still possess limitations related to immune responses and biodegradability [6,7]. Naturally derived materials, on the other hand, have been explored with the idea that they could better resemble the native environment of cardiac cells [8,9], especially those based on the extracellular matrix (ECM) obtained from the decellularization of cardiac tissues (dECM) [10,11,12]. An interesting feature of dECM patches is that, because of their biomimetic nature, they can be repopulated with human mesenchymal stromal cells (hMSCs) to enhance their regenerative potential [3,13]. Although such developed cardiac patches have shown good outcomes, they still present certain limitations, for instance, their inadequate mechanical properties (e.g., low stiffness) and the lack of patient-specific shapes [14,15]. Thus, there is a need for bioprintable cell-laden hydrogels (bioinks) to develop customized patches exhibiting enough mechanical strength.

In the present work, we describe an optimized protocol to produce cardiac hydrogels from the enzymatic digestion of decellularized porcine myocardium (cECM) that can be loaded with cells, and which are bioprintable. The bioink is generated in a way that can be fabricated and bioprinted without the need to mix the cECM with other biomaterials or incorporate external crosslinkers. The mechanical properties of the developed hydrogel were assessed by rheometry and its ultrastructure by imaging with a Scanning Electron Microscope. The cECM bioink (loaded with human-bone-marrow-derived mesenchymal stem cells -hBM-MSCs) was bioprinted by using a two-printhead strategy with a sacrificial material. The behavior of the cells when cultured in 3D within the developed scaffolds was then studied by immunostaining and contraction assays.

## 2. Results and Discussion

### 2.1. Macro- and Ultra-Structure of the Hydrogels Developed from the Digested cECM

**Figure 1:**
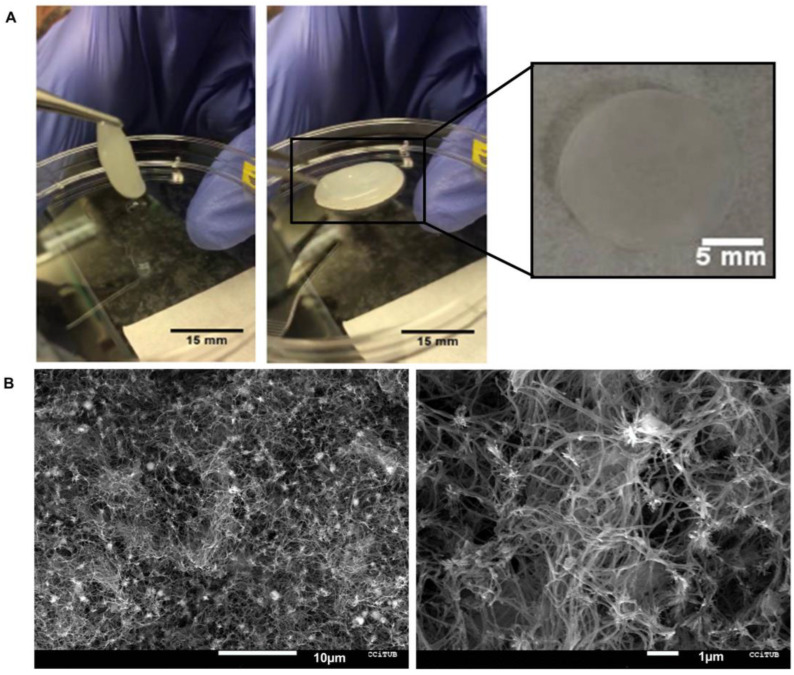
Structural characterization of the cECM hydrogels. Macro image of the cECM hydrogel, showing a homogeneous structure and its manipulation with tweezers (**A**). SEM images of the cECM hydrogel (**B**).

The hydrogels showed a homogeneous structure with no macroscopically visible fiberboard clusters. Structures formed with the developed hydrogels showed enough strength to be manipulated with tweezers without breaking and with the capability of recovering the original shape after manipulation (Figure 1A). This improvement with respect to previously reported studies is achieved by working at higher powder concentration and by optimizing the digestion time (as compared with the common protocol originally published by Freytes and coworkers [16]). Thanks to such improvement in mechanical strength, the 3D-bioprinted structures can be manipulated with surgical tools so they could be implanted in vivo by using a bio-glue. This aspect represents an important advance in the case of patches developed from ECM-derived cardiac bioinks. Indeed, to have the required strength, previously reported materials presented weaker mechanical properties, so they should be mixed with other biomaterials [17] not naturally included in the native tissue or additionally crosslinked [18].

Regarding to ultrastructure, SEM images of the hydrogels showed a fibrillary structure as expected (Figure 1B). The average diameter of the fibers of the cECM hydrogel was 126 ± 8 nm. These data agree with the diameter of tropocollagen fibers [19], which is consistent with collagen I fibrils, reported to be in the 100 nm diameter range [20]. The structure observed in cECM hydrogels is closer to that of the native ECM compared with previous works [21], showing fibers with a diameter slightly below that of the collagen present in the ECM. As can be observed in Figure 1B, the structures obtained with the developed bioink presented a distribution of the fibers resembling that of the original cardiac ECM [22,23]. Moreover, thanks to the small diameter of the fibers, the hydrogel presented a high contact area [24], which has been reported to improve cell attachment and proliferation [25,26].

### 2.2. Rheological Properties of the cECM Hydrogels Depending on the Pepsin Digestion Time

**Figure 2:**
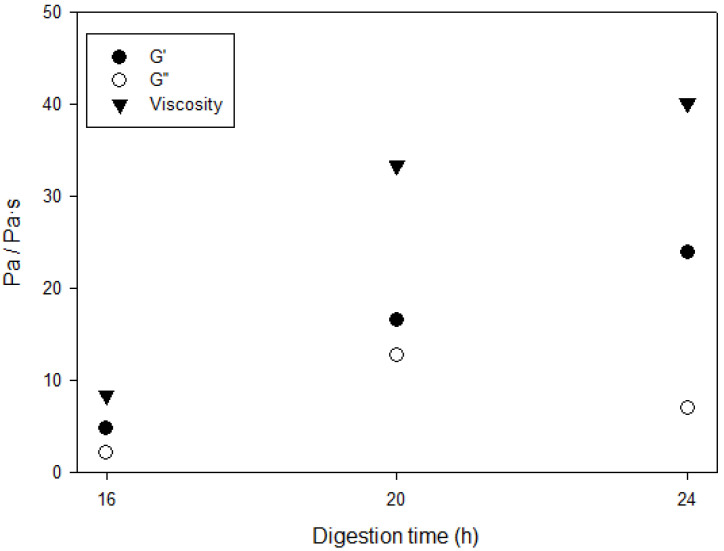
Rheological properties of the cECM hydrogels as a function of the pepsin digestion time (*N* = 2).

The rheological properties of the cECM hydrogels depended on the digestion time (Figure 2). Gelation started gradually, being the hydrogel digested for 24 h the one showing the highest storage modulus (G′ = 23.9 ± 10.6 Pa), and the hydrogel digested for 16 h, the one with the lowest storage modulus (G′ = 4.8 ± 1.4 Pa). Of note, for digestion times above 24 h, the cECM did not reach gelation, in keeping with previous reports regarding other natural, organ-derived hydrogels, such as lung extracellular matrix or type I collagen hydrogels [27].

Thus, 24 h was the chosen digestion time for the bioinks used in the rest of the experiments in the present work. The required digestion time is highly dependent on the particle size obtained after decellularization and cryogenic milling. If the digestion time is not long enough, big particles cannot be fully solubilized, while if the time is higher than optimal, pepsin may start to degrade certain proteins that are crucial for hydrogel formation.

### 2.3. 3D Cultures of hBM-MSCs

**Figure 3:**
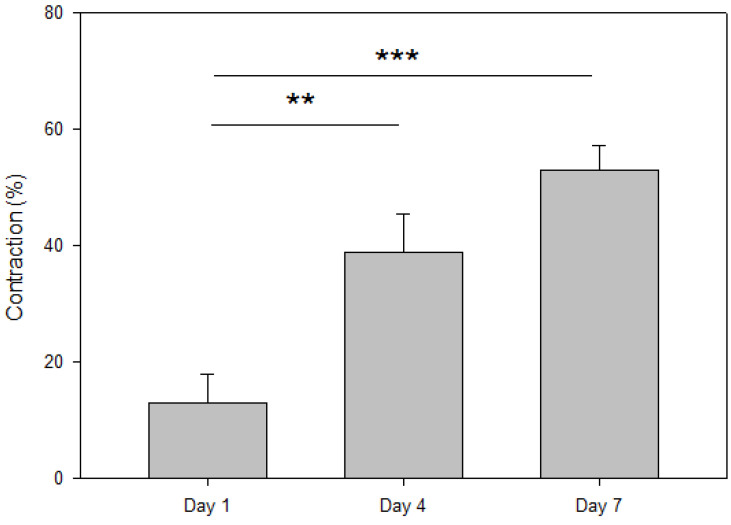
Contraction of the 3D-cultured cECM hydrogels (hBM-MSC) with respect to acellular ones (control) for 1, 4 and 7 days. **: *p* < 0.01; ***: *p* < 0.001.

When cultured in 3D, hBM-MSCs contracted the cECM hydrogels, as measured via the hydrogel contraction assay (Figure 3). As expected, the shrinkage of the structures increased with the culture time. After 1 day of 3D culture, the measured area of the cECM hydrogels with hBM-MSCs was reduced to 158.5 ± 8.3 mm^2^ (12.9 ± 4.5 percent of contraction; *p* = 0.076). At day 4, the measured value was 111.2 ± 11.2 mm^2^ (38.9 ± 6.2 percent of contraction; *p* = 0.005), and after 7 days in culture, the area was 85.7 ± 7.4 mm^2^ (52.9 ± 4.1 percent of contraction; *p* < 0.001). 

The contraction observed in the cell-laden structures indicated an active crosstalk between the cECM hydrogel matrix and the cells. The concentration of cells used in the present study, which is in the lower range (2.5 × 10^5^–5 × 10^5^ cells/mL) recommended in the seminal paper by Freytes and coworkers for stem cell-laden cardiac hydrogels [16], seemed to be enough for this type of cells to interact with their surrounding ECM. Nevertheless, several effects could be overlapping, explaining the observed contraction, as cells can be effectively pulling the fibers of the structure while, at the same time, degrading matrix proteins by secreting metalloproteinases. Previous studies conducted with fibroblasts and stromal cells cultured in collagen matrices have shown that the combination of both factors strongly depends on structural mechanics and other stimuli that may alter cell contractility [28,29,30]. Although it is out of the scope of the present study, further work should be carried out to determine which of the mechanisms is dominating for this kind of cells in cECM hydrogels. 

**Figure 4:**
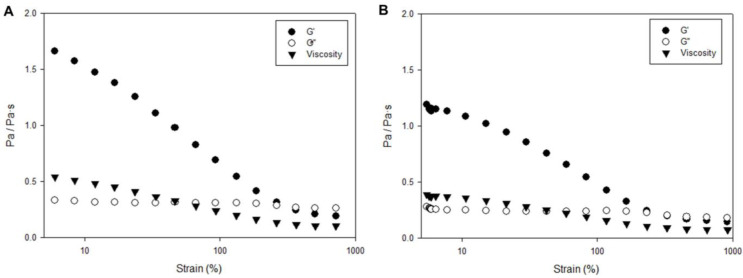
Comparison of the storage modulus (G′, G″ and viscosity) in acellular (**A**) and cellular hydrogels (**B**) in a representative example.

To better understand these cell–matrix interactions, the alteration of the mechanical properties of the structures due to the cell culture was studied via rheometry. As shown in Figure 4, cell-laden structures were softer when compared with the acellular ones. As expected, values for the shear modulus and viscosity decreased with the applied strain. Measured rheological properties were in the same range of values previously reported for other cECM hydrogels [15]. Interestingly, cell-laden hydrogels seemed to be softer than acellular ones as shown in the representative example in Figure 4. Nevertheless, these experiments have the limitation that the size of cell-laden structures varies from replicates due to contraction so an extensive analysis of this aspect would be extremely complex (due to physical limitations of the rheometer) and it is out of the scope of this work. Even in this case, an explanation for the observed softening of the structures could be that cells are generally softer than their surrounding ECM or by the fact that cells are degrading the ECM where they are cultured.

**Figure 5:**
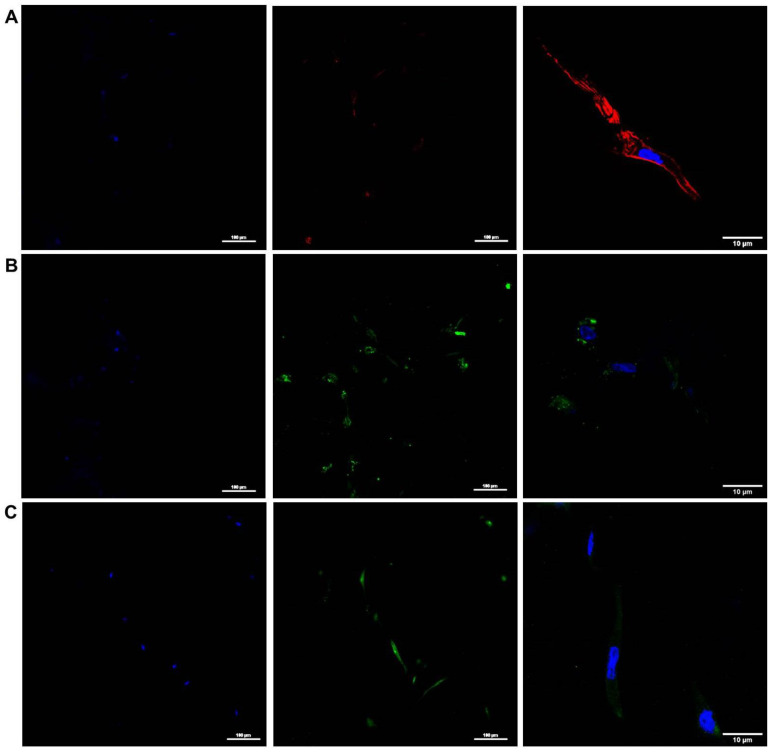
Immunostaining of hBM-MSC 3D-cultured in the cECM hydrogels for 7 days. (**A**) Phalloidin (red). (**B**) α-SMA (green). (**C**) Cx43 (green). Left and center images are low-magnification (20×) with split channels, while the nucleus is counter-stained in blue; right images are high-magnification (100×) images.

Immunofluorescence images of 3D cultured hBM-MSCs for 7 days can be observed in Figure 5. Cells were stained for Connexin 43 (Cx43) and α-Smooth muscle actin (α-SMA) in green. The actin filaments of the cytoskeleton and the nuclei were also stained. Together with high-magnification images to show single-cell morphology, low-magnification images reveal that there are several cells positive for the different stainings. 

Even with the difficulties of imaging cells within hydrogels (cells are not as well aligned with the imaging plane as they are when imaged on top of plane surfaces), data from Figure 5 illustrate that cells presented a well-formed cytoskeleton with the characteristic spindle shape of MSCs. Additionally, immunostaining revealed that cells expressed Cx43 and α-SMA after 7 days of 3D culture within the developed scaffolds.

The observed results indicate that the cells were able to remain alive when cultured in the developed cardiac patches built with cECM bioinks, showing a cytoskeleton-spindle-like morphology typical of MSCs [31]. It has been previously proven that hMSCs have the ability to release paracrine factors and extracellular vesicles (EVs) exhibiting immunomodulatory, anti-inflammatory, and antimicrobial effects [32,33,34,35,36]. The proliferation and/or differentiation of the cells within the developed dECM hydrogels are not studied in the present work. Proliferation has not been generally observed in previous studies in short periods of culture (1 week), while differentiation was not expected to occur. Indeed, hBM-MSCs were maintained in culture by using manufacturer-recommended medium and supplements, which are designed to keep the stemness of the cultured cells. Although, the presented data show cell survival together with the positive expression of Cx43 and α-SMA (which indicated cell–cell and cell-matrix interactions, which have been shown to play an important role in the physiology of the heart [37,38] and its ECM remodeling [39,40]) it was shown that the developed bioinks at the cell concentration used are suitable for the development of hMSC-loaded cardiac patches (although it would be interesting to test lower and higher concentrations in future works). 

Bioinks developed exclusively from the digestion of decellularized ECM should be printed in liquid phase, hence a support material is needed (F127 in our case), which should be removed after the crosslinking of the final structures. This aspect represents a limitation if complex shapes must be developed, but it is well suited for developing a customized cardiac patch. Moreover, as the bioink is printed in liquid phase, the pressure required is quite low (lower that the one exerted on the cells when manipulated with a standard laboratory pipette), thus fastening the test phase of new developments as they can be carried out with standard labware prior to testing in with the bioprinter.

Previous studies have reported the use of pericardium dECM to engineer patches for the treatment of myocardial infarction [3]. Indeed, rat dECM patches have successfully replaced the right ventricular outflow tract defect in Lewis rat models, showing no differences between the area in contact with the patch and the healthy ventricles [41]. Moreover, patches have also shown signs of neovascularization and nerve sprouting in the infarcted area that was in contact with either human pericardial or porcine myocardial dECM scaffolds [13]. The results presented herein extend the potential of cardiac patches based on dECM, as they open the door to developing custom-shaped grafts using 3D bioprinting technology [42].

The idea of customizing the shape of the cardiac patches started when Pati et al. [43] printed three-dimensional cardiac tissue with cECM hydrogel made from porcine dECM and human-adipose-derived MSCs, achieving high cell viability. Jang et al. [44] added Vascular Endothelial Growth Factor (VEGF) and MSCs to a porcine dECM pregel to promote a rapid vascularization of cell-laden heart cECM bioink. In both cases, polycaprolactone (PCL, a synthetic polymer) was used to provide mechanical support to the structure, due to the weak mechanical properties of their cECM hydrogel alone. Other approaches used crosslinkers [45,46,47] and biomaterials from natural and synthetic origins (such as silk, genipin [18], or gelatin methacrylate-GelMA [14]) with the same objective of obtaining patches with enough strength to be manipulated with surgical tools and hence for in vivo application. The solution achieved in the present work by optimizing the digestion of cECM overcomes those previous limitations, as there is no need to use a complementary biomaterial or to crosslink the patches after 3D bioprinting. 

## 3. Conclusions

The use of MSCs for the repair of the infarcted zones of the heart is a promising therapy, with the release of paracrine factors as the main mechanism of repair involved. The use of MSC-laden grafts in the form of myocardial patches to be implanted in the heart represents a major advance in this field, since the therapeutic effect of the cells can be sustained over time when using this strategy. Nevertheless, MSCs are highly sensitive to the physico-chemical properties of their ECM microenvironment. Therefore, it is important to mimic the characteristics of the ECM as much as possible when engineering a therapeutic graft. Among all possible procedures, the use of cardiac ECM obtained via native tissue decellularization as a biomaterial to develop the grafts is one the most promising nowadays, because the scaffold has components from the ECM exclusively. In this work, we have developed a novel cardiac ECM bioink (digested decellularized myocardial ECM mixed with hBM-MSCs and bioprinted in the pregel phase) in such a way that the obtained patches can be manipulated with surgical tools, and thus applied in vivo, not requiring the use of additional biomaterials or crosslinkers. Moreover, the printability (using a two-nozzle system with a supporting material) of the bioink allows for custom-shaped patch development. The morphology and expression of relevant markers of hMSCs cultured within the developed scaffolds showed effective cell–cell and cell–matrix interactions after a 1-week culture. However, further in vivo studies in animal models are required to ascertain the therapeutic effect of these cell-cultured scaffolds. Nevertheless, the preliminary results we show herein, together with results we previously reported for MSCs cultured within lung ECM hydrogels [24], are promising and suggest that the cECM bioink presented in this work could be an excellent material to develop customized patches for cell therapy in cardiac repair. 

## 4. Materials and Methods

All the reagents employed were obtained from Sigma Aldrich (Saint Louis, MO, USA) unless otherwise specified.

### 4.1. Preparation of the cECM Bioinks from the Decellularization of Porcine Myocardia

Porcine hearts were obtained from a local slaughterhouse and then washed with deionized water before freezing them at −80 °C to promote cell lysis and storage until further processing. The decellularization method was adapted from a protocol developed for human hearts [46], with slight modifications. Briefly, hearts were thawed to room temperature (RT), and the left ventricular myocardium was sectioned into 1 × 1 × 1 cm^3^ cubes and frozen for further cryo-sectioning into 300 µm-thickness slices using a cryostat (HM 560, Thermo Fisher Scientific, Waltham, MA, USA). The resulting myocardial slices were then decellularized via sequential immersion in lysis solution (10 mM Tris, 0.1% *w*/*v* ethylenediaminetetraacetic acid (EDTA), pH 7.4 in dH_2_O, 2 h at RT), 0.5% sodium dodecyl sulfate (SDS) (6 h at RT), and fetal bovine serum (FBS) (3 h, 37 °C) with intermediate washes in phosphate-buffered saline (PBS) 1x. At the end of the decellularization process, the slices were drained and stored at −80 °C. To produce the cECM powder, slices were freeze-dried for 48 h (Lyoquest-55 Plus, Telstar, Terrassa, Spain) and pulverized into a micrometric powder using a cryogenic miller (6775 Freezer/Mill, SPEX, Metuchen, NJ, USA). 

**Figure 6:**
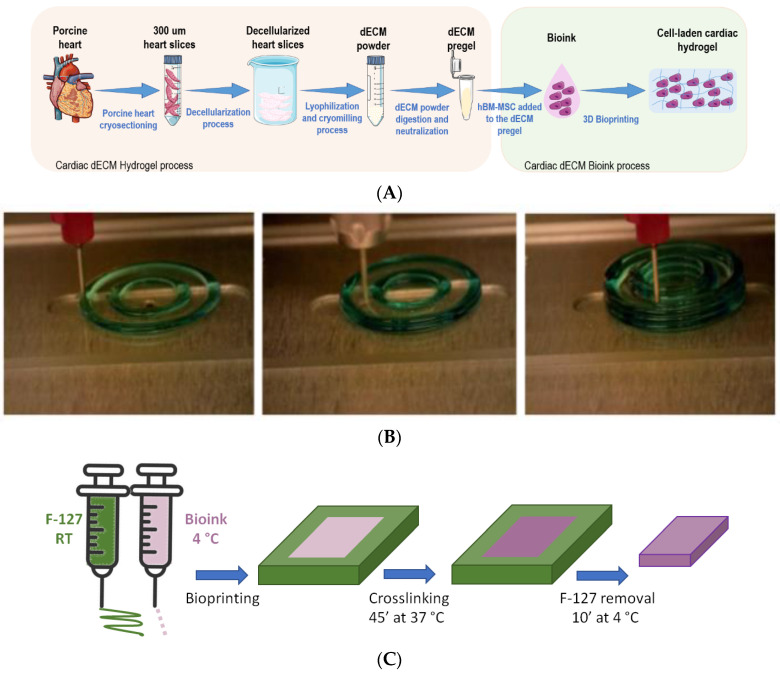
(**A**) Schematic description of the process followed to obtain cECM pregel and the bioink. (**B**) Bioprinting process using F-127 as supportive/sacrificial layer (in green), adapted from [24]. (**C**) Schematic of the technique used for the bioprinting the cell-laden pregel, crosslinking the structure and then removing the supportive layer of F-127.

The resulting cECM powder was digested at a concentration of 20 mg/mL in a 0.1 M HCl solution with pepsin from porcine gastric mucosa (1:10 concentration) under magnetic stirring at RT for different times (for experiments other than rheology, 24 h digestion was chosen as this time and resulted in the highest storage modulus). After digestion of the ECM (where pH never rose above 3), the pregel solution was stabilized to pH = 7.4 ± 0.4 using 1 M NaOH (1:10 *v*/*v* respect to the HCl volume) and PBS 10x (1:10 *v*/*v* respect to the neutralized volume). For 3D bioprinting, after loading the pregel with the cells (Figure 6A, Section 4.4 of the present work for more details) the dual-printhead method described in [27] was used as schematically represented in Figure 6C. Briefly, one printhead of the 3D bioprinter (3Ddiscovery, RegenHU, Switzerland, installed inside a safety laminar flow cabinet) was filled with the pregel and maintained at 4 °C to prevent gelification. A secondary printhead was filled with Pluronic F-127 gel at room temperature (RT). Structures were then bioprinted by alternatively printing an F127 layer (40% *v*/*v* in PBS 1x, printed at ~4.5 atm with a needle of 250 µm of diameter—Nordson EFD, Westlake, OH, USA), which served as a template, and a pregel layer to form the desired shape. As the pregel is maintained in liquid phase, the pressure exerted was just above atmospheric pressure. At the end of the bioprinting process, scaffolds were incubated at 37 °C for 45 min to crosslink the cell-laden cECM hydrogels. Finally, the Pluronic was dissolved by immersing the developed structures in culture media at 4 °C for 10 min.

### 4.2. Ultrastructural Characterization by Scanning Electron Microscopy

The ultrastructure of the cECM hydrogel scaffolds was visualized with a JSM-6510 (JEOL, Tokyo, Japan) scanning electron microscope (SEM). cECM scaffolds of 20 × 9 × 3 mm^3^ were produced via 3D bioprinting casting 

The 3D scaffolds were fixed in 4% paraformaldehyde (PFA) in PBS for 48 h and then washed three times with 0.1 M phosphate buffer (PB). Next, the samples were incubated in 4% osmium tetroxide for 90 min and then rinsed with deionized water. Subsequently, samples were dehydrated by washing them with ethanol 80% (×2), 90% (×3), 96% (×3), and 100% (×3) and preserved in absolute ethanol at 4 °C until critical point drying (Autosamdri-815 critical point dryer, Tousimis, Rockville, MD, USA). Samples were then carbon coated and mounted using conductive adhesive tabs (TED PELLA, Redding, CA, USA). Imaging was performed by using an SEM (JSM-6510, JEOL, Tokyo, Japan) at 15 kV.

The diameter of the fibers was assessed following the method developed in [47]. Briefly, 10 fibers of three different zones of each sample were randomly selected, and their diameter was computed with ImageJ Software v1.53 (National Institute of Health, Bethesda, MD, USA).

### 4.3. Hydrogel Rheology

The rheology of the developed bioinks was measured using a Haake RheStress1 rheometer (Thermo Fisher, MA, USA) with a 35 mm serrated parallel plate. The storage (*G′*) and loss (*G″*) moduli were measured, and the modulus of the complex viscosity (η*) was calculated by using Equation (1) for a given frequency ω.

(1) $$\eta^{*}=\eta^{\prime }-{i\eta }^{\prime \prime }=\frac{G^{\prime \prime }}{\omega }-\frac{{iG}^{\prime }}{\omega }$$

Hydrogels were digested for 16 h, 20 h, 24 h, and 28 h. The gelation kinetics of the acellular hydrogels was assessed by loading a pregel solution onto a Peltier plate at 4 °C for 10 min at 0.628 rad/s frequency (0.1 Hz). The temperature was kept constant for 10 min, subsequently increased to 37 °C, and kept constant for 15 min.

### 4.4. 3D Cell Culture of hBM-MSCs

Human bone marrow mesenchymal stromal cells (ATCC, Manassas, VA, USA) were expanded in tissue culture plates by following the manufacturer’s instructions. Cells in passages 3–5 were used for conducting all the experiments in this study. 

The 3D cultures were prepared by cooling the digested ECM solution to 4 °C in the fridge and then maintaining it in ice prior to neutralization by using ice-cold NaOH and PBS 10X as previously described. Once the pregel was at 4 °C and at physiological pH, it was mixed with the cells resuspended in ice-cold culture media (2.5 × 10^6^ cells/mL) at a relation 10:1 *v*/*v*. To form the cell-laden hydrogel structures, the resulting mixture was then incubated at 37 °C for 45 min to form disk-shaped structures of 1.9 cm^2^ of surface area. The final cell concentration in the scaffolds was 2.5 × 10^5^ cells/mL. The cell-laden structures were cultured for 7 days, changing the medium every 3 days. 

The diameter of cell-laden structures was measured on days 1, 4, and 7 after cell seeding. cECM hydrogels were imaged with a high-resolution camera fixed to a tripod after aspirating the culture media to avoid image errors due to the movement of the structure. The surface area of the 3D cultures was quantified from the acquired images by using ImageJ Software, and the contraction was calculated as the percentage of reduction with respect to the corresponding acellular controls [27].

The influence of the cells on the rheological properties once the structures were gellified was assessed for the cultures at 7 days. Then, rheometry on the cell-laden hydrogels (24 h of pepsin digestion) was characterized at 37 °C by using an amplitude sweep from 5% (which showed to be the deformation for the cell-laden hydrogels when applying the lowest value for tension that the equipment allowed) to 1000% at a frequency of 3.14 rad/s (0.5 Hz). 

For the immunohistochemical analysis of the cells cultured in 3D within the cardiac bioinks, cell-laden hydrogels at day 7 were immersed in Optimal Cutting Temperature (OCT) and frozen at −80 °C. Thin hydrogel slices (≈12 µm) were obtained via cryo-sectioning (HM 560, Thermo Fisher Scientific, MA, USA) and placed on top of positively charged glass slides. OCT was removed by thawing and washing the samples in PBS 1X solution at room temperature. Subsequently, cells were fixed with 4% PFA for 15 min. Primary and secondary antibodies were incubated overnight and for 2 h at 37 °C. Nuclei were stained with Hoechst 33342 (Thermo Fisher Scientific, MA, USA) for 15 min. Primary antibodies employed were anti-Cx43 (ab11370, abcam, Cambridge, UK) and anti-αSMA (ab32575, abcam, Cambridge, UK). Secondary antibodies used were Alexa 488 goat anti-rabbit (ab150081, abcam, Cambridge, UK) and Alexa 488 goat anti-mouse antibody (ab150117, abcam, Cambridge, UK). Images were acquired with a Nikon D-Eclipse Ci confocal microscope with a 20× and 100× Plan Apo objectives (Nikon, Tokyo, Japan). Usual controls for immunostaining (e.g., without the primary antibody or without the secondary antibody) were performed to ensure non-specific staining in the experiments.

### 4.5. Statistical Analysis

Data are expressed as mean ± SE. For the digestion time and contraction analyses, one-way analysis of variance (ANOVA), followed by the Holm–Sidak’s post hoc test, was conducted, while paired t-test was performed for rheological characterization. Statistical significance was considered at *p*-values < 0.05. 

## Data Availability

The datasets generated for this study are available on justified request from the corresponding author.
